# *Saccharomyces cerevisiae* Modulates Immune Gene Expressions and Inhibits ETEC-Mediated ERK1/2 and p38 Signaling Pathways in Intestinal Epithelial Cells

**DOI:** 10.1371/journal.pone.0018573

**Published:** 2011-04-04

**Authors:** Galliano Zanello, Mustapha Berri, Joëlle Dupont, Pierre-Yves Sizaret, Romain D'Inca, Henri Salmon, François Meurens

**Affiliations:** 1 Société Industrielle Lesaffre, Lesaffre Feed Additives, Marcq-en-Baroeul, France; 2 Institut National de la Recherche Agronomique (INRA), UR1282, Infectiologie Animale et Santé Publique, Nouzilly (Tours), France; 3 Institut National de la Recherche Agronomique (INRA), UMR85, Physiologie de la Reproduction et des Comportements, Nouzilly (Tours), France; 4 Département des microscopies, plate-forme R.I.O de microscopie électronique, Université François Rabelais, Tours, France; University of California Merced, United States of America

## Abstract

**Background:**

Enterotoxigenic *Escherichia coli* (ETEC) infections result in large economic losses in the swine industry worldwide. ETEC infections cause pro-inflammatory responses in intestinal epithelial cells and subsequent diarrhea in pigs, leading to reduced growth rate and mortality. Administration of probiotics as feed additives displayed health benefits against intestinal infections. *Saccharomyces cerevisiae* (*Sc*) is non-commensal and non-pathogenic yeast used as probiotic in gastrointestinal diseases. However, the immuno-modulatory effects of *Sc* in differentiated porcine intestinal epithelial cells exposed to ETEC were not investigated.

**Methodology/Principal Findings:**

We reported that the yeast *Sc* (strain CNCM I-3856) modulates transcript and protein expressions involved in inflammation, recruitment and activation of immune cells in differentiated porcine intestinal epithelial IPEC-1 cells. We demonstrated that viable *Sc* inhibits the ETEC-induced expression of pro-inflammatory transcripts (IL-6, IL-8, CCL20, CXCL2, CXCL10) and proteins (IL-6, IL-8). This inhibition was associated to a decrease of ERK1/2 and p38 MAPK phosphorylation, an agglutination of ETEC by *Sc* and an increase of the anti-inflammatory PPAR-γ nuclear receptor mRNA level. In addition, *Sc* up-regulates the mRNA levels of both IL-12p35 and CCL25. However, measurement of transepithelial electrical resistance displayed that *Sc* failed to maintain the barrier integrity in monolayer exposed to ETEC suggesting that *Sc* does not inhibit ETEC enterotoxin activity.

**Conclusions:**

*Sc* (strain CNCM I-3856) displays multiple immuno-modulatory effects at the molecular level in IPEC-1 cells suggesting that *Sc* may influence intestinal inflammatory reaction.

## Introduction

Enterotoxigenic *Escherichia coli* (ETEC) is a major cause of intestinal infection in piglets inducing diarrhea, reduced growth rate and mortality leading to economic loss [1]. Pathogeny of ETEC is characterized by its adhesion to the intestinal epithelial cells (IEC) through adhesins which interact with their specific receptors localized on the brush border membrane [2], [3], [4]. Following jejunal and ileal mucosa colonization, ETEC strains secrete several enterotoxins, including the heat-labile enterotoxin (LT), the heat-stable enterotoxin (STa and/or STb), and the enteroaggregative *E. coli* heat-stable enterotoxin 1 (EAST1) [5], [6]. These enterotoxins cause perturbation of hydroelectrolytic secretions in the small intestine resulting in diarrhea [5].

ETEC strains expressing the F4 *fimbriae* are involved in neonatal and post-weaning diarrhea [1]. ETEC F4^+^ infections mainly occur during the first week after weaning in piglets expressing the F4 receptor on the intestinal brush border [7]. The weaning-related stress, the dietary changes and the immaturity of the immune system are several factors contributing to the disease severity [1]. ETEC F4^+^ strains represent the most prevalent form of bacterial infection in piglets [1], [8] and an increase in incidence of ETEC-associated diarrhea was observed worldwide [1]. Furthermore, antibiotic growth promoters were prohibited in the European Union since 2006 (IP/05/1687) and antibiotic-multiresistant ETEC isolates have been identified [9], [10], [11]. Consequently, new prophylactic and/or therapeutic strategies should be developed to protect piglets from ETEC infection. The interest in using probiotic microorganisms such as live yeasts to prevent gastrointestinal diseases in farm animals has increased significantly in the last decade worldwide. *Saccharomyces cerevisiae* variety *boulardii* (*Sb*) has been shown to provide intestinal protection against various enteric pathogens [12]. Indeed, *Sb* protected the host through multiple mechanisms such as inhibition of pathogen adhesion [13], neutralization of bacterial virulence factors [14], maintenance of epithelial barrier integrity [15], decrease of pathogen-associated inflammation [16] and stimulation of the immune system [17]. Regarding *Sb* effects on pathogen-associated inflammation, this yeast has been shown to modulate pro-inflammatory signaling pathways leading to the inhibition of mitogen-activated protein kinases (MAPK) and nuclear factor NF-κB activition in IEC [16], [18]. Because IEC play a key role in regulating innate and adaptive immune responses of the gut [19], several studies have evaluated yeast probiotic effects on these cells [15], [16], [18], [20], [21], [22], [23]. Epithelial cells protect the intestine through different mechanisms such as barrier function, mucus secretion, antibacterial peptide synthesis, cytokine and chemokine secretions [19]. IEC detect pathogen-associated molecular patterns (PAMPs) through their pathogen recognition receptors (PRR) and then secrete cytokines and chemokines that activate pro-inflammatory signaling pathways and direct the migration of various effector cells involved in innate and adaptive immunity [24]. However, inflammatory responses induced by enteric pathogens can lead to dysregulation of IEC signaling, disruption of membrane barrier integrity, enhancement of pathogen translocation and disease [25]. With their pivotal role in the gut homeostasis, IEC are particularly relevant to assess yeast immuno-regulatory effects.

*Saccharomyces cerevisiae* (*Sc*) is non-commensal and non-pathogenic yeast used in food industry as brewer and baker's yeast. *Sc* and *Sb* are members of the same yeast species [26] but they present some genetical, metabolical and physiological differences [27], [28]. *Sc* (strain CNCM I-3856) is a probiotic yeast studied for its beneficial effects on animal growth, host immune function and inhibition of *Salmonella spp.* adhesion [29], [30]. Furthermore, *Sc* (strain CNCM I-3856) has been shown to decrease inflammation in a mouse model of chemically-induced colitis [31], to reduce digestive discomfort and abdominal pain in IBS patients [32] and to exert *in vitro* antagonist effect against *E. coli* O157:H7 [33]. In the current study, we use an *in vitro* model of differentiated porcine intestinal epithelial IPEC-1 cells co-cultured with *Sc* (strain CNCM I-3856) and F4^+^ ETEC (strain GIS26). IPEC-1 cells provide a relevant model since F4^+^ ETEC has been shown to bind IPEC-1 cells which express cytokines and chemokines after ETEC stimulation [34], [35]. In addition, the ETEC strain GIS26 has been shown to infect newly weaned piglets (Verdonck et al. 2002). Few data are available regarding yeast immuno-modulatory effects in porcine IEC exposed to ETEC. These data showed that *Sc* and *Sb* inhibited IL-1α transcript expression in non-differentiated IPEC-J2 cell line [36]. Consequently, using differentiated IPEC-1 cells, we investigated in this report whether *Sc* modulates gene expressions and signaling pathways involved in inflammation, recruitment and activation of immune cells. Then, we assessed whether *Sc* could prevent the disruption of the membrane barrier integrity induced by ETEC.

## Results

### *Saccharomyces cerevisiae* (strain CNCM I-3856) and ETEC (strain GIS26) modulate differently immune gene expression in differentiated IPEC-1 cells

**Figure 1:**
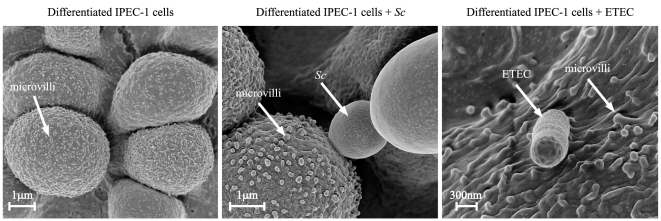
Interaction of *Saccharomyces cerevisiae* or ETEC with differentiated IPEC-1 cells. Differentiated IPEC-1 cells were cultured overnight with *Sc* (3×10^6^ yeasts/well) or exposed to ETEC (3×10^7^ CFU/well) for 45 min. IPEC-1 cells were then assessed to scanning electron microscopy. (A) Differentiated IPEC-1 cells expressed microvilli on their surface, (B) *Sc* interacts with differentiated IPEC-1 cells, (C) ETEC interacts with differentiated IPEC-1 cells.

In this work, we aimed to compare the effects of *Sc* and ETEC on IPEC-1 gene expressions involved in inflammation, innate and adaptive immunity. As illustrated by scanning electron microscopy, differentiated IPEC-1 cells displayed microvilli and both *Sc* and ETEC interact with these cells (Figure 1). Characterization of *Sc* and ETEC immuno-modulatory effects was first assessed by analysis of transcript expressions. The analysed genes are presented in Table 1. ETEC increased significantly the mRNA expression *(p<0.01)* of the pro-inflammatory cytokines TNF-α (x 3075.6), IL1-α (x 46.9), IL-6 (x 7.8), the pro-Th2 cytokine IL-5 (x 2.6), and the chemokines CCL20 (x 5726.1), CXCL2 (x 857.4), IL-8 (x 670.2), CXCL10 (x 7.1) and CXCL12 (x 3) (Table 2). In contrast, *Sc* did not significantly up-regulate these transcripts but increased the expression *(p<0.01)* of the pro-Th1 cytokine IL-12p35 (x 10.7), the chemokine CCL25 (x 2.7), the anti-inflammatory nuclear receptor PPAR-γ (x 2.6), the mucine MUC1 (x 2.21) and decreased the mRNA expression of the pro-Th2 cytokine BAFF (÷ 2.5, *p<0.01*) (Table 2). Thus, this result shows that *Sc* and ETEC display different modulatory effects on transcripts involved in both inflammatory and immune responses.

**Table 1:** Primer sequences, annealing temperatures of primer sets (°C), expected PCR fragment sizes (bp) and accession numbers or references.

Primer name	Primer sequence	Annealing temperature(s) (°C)	PCR product (bp)	Accession number or reference
**APRIL/**TNFSF13	S: TGCTCACCCGTAAACAGAAGAS: TAAACTCCAGCATCCCAGAC	60	172	Meurens *et al.*, 2009
**BAFF/**TNFSF13B	S: GAGAGCAGCTCCATTCAAAGAS: GCATGCCACTGTCTGCAATC	60	103	Meurens *et al.*, 2009
**CCL2/**MCP-1	S: GTCACCAGCAGCAAGTGTCAS: CCAGGTGGCTTATGGAGTC	60	112	Meurens *et al.*, 2009
**CCL17/**TARC	S: TGCTGCTCCTGGTTGCTCTCAS: ATGGCGTCCCTGGTACACTC	67	169	Bruel *et al.*, 2009
**CCL20/**MIP3 alpha	S: GCTCCTGGCTGCTTTGATGTCAS: CATTGGCGAGCTGCTGTGTG	66	146	Meurens *et al.*, 2009
**CCL22/**MDC	S: GTCCTCCTTGCTGTGATACAS: CTCGGTCCCTCAAGGTTAG	60	184	DB798783
**CCL25/**TECK	S: ACCTGCCTGCTGTGATATTCAS: TCCGATTGTCCAGGATCTTC	62	105	NM_001025214
**Cdx-1**	S: ACAGCCGCTATATCACCATCAS: GTTCACTTTGCGCTCCTTTG	60	116	ENSFM00600000921619 http://ensembl.org
**Cdx-2**	S: CAGTCGCTACATCACCATTCAS: GCTGTTGCTGCAACTTCTTC	60	137	GU_17420
**CXCL2/**GRO beta	S: TGCTGCTCCTGCTTCTAGTGAS: TGGCTATGACTTCCGTTTGG	60	171	Meurens *et al.*, 2009
**CXCL10/**IP-10	S: CCCACATGTTGAGATCATTGC AS: CATCCTTATCAGTAGTGCCG	60	168	Meurens *et al.*, 2009
**CXCL12/**SDF-1	S: TGCCTCAGCGATGAGAAACAS: GGGTCAATGCACACTTGTC	58	173	AY312066
**HMBS2**	S: AGGATGGGCAACTCTACCTGAS: GATGGTGGCCTGCATAGTCT	58	83	Nygard *et al.*, 2007
**HPRT-1**	S: GGACTTGAATCATGTTTGTGAS: CAGATGTTTCCAAACTCAAC	60	91	Nygard *et al.*, 2007
**IFN gamma**	A: GCTCTGGGAAACTGAATGACAS: TCTCTGGCCTTGGAACATAG	60	167	Meurens *et al.*, 2009
**IL-1 alpha/LAF**	S: CCCGTCAGGTCAATACCTCAS: GCAACACGGGTTCGTCTTC	60	170	NM_214029
**IL-4/BCGF**	S: CAACCCTGGTCTGCTTACTGAS: CTTCTCCGTCGTGTTCTCTG	65	173	Meurens *et al.*, 2009
**IL-5/EDF**	S: TGGAGCTGCCTACGTTAGTGAS: TCGCCTATCAGCAGAGTTCG	64	105	Meurens *et al.*, 2009
**IL-6/IFN beta 2**	S: ATCAGGAGACCTGCTTGATGAS: TGGTGGCTTTGTCTGGATTC	62	177	Meurens *et al.*, 2009
**IL-8/**CXCL-8	S: TCCTGCTTTCTGCAGCTCTCAS: GGGTGGAAAGGTGTGGAATG	62	100	Meurens *et al.*, 2009
**IL-10/**B-TCGF	S: GGTTGCCAAGCCTTGTCAGAS: AGGCACTCTTCACCTCCTC	60	202	NM_214041
**IL-12p35**	S: GGCCTGCTTACCACTTGAACAS: GCATTCATGGCCTGGAACTC	64	180	Meurens *et al.*, 2009
**IL-13**	A: TGGCGCTCTGGTTGACTCTGAS: CCATGCTGCCGTTGCATAGG	67	159	Meurens *et al.*, 2009
**IL-17A/**CTLA-8	A: CCAGACGGCCCTCAGATTACAS: CACTTGGCCTCCCAGATCAC	66	103	Meurens *et al.*, 2009
**IL-23p19**	S: CTCCTTCTCCGCCTCAAGATCC AS:TTGCTGCTCCATGGGCGAAGAC	70	82	Meurens *et al.*, 2009
**IL-33**	S: AGCTTCGCTCTGGCCTTATCAS: GCTGACAGGCAGCAAGTACC	63	126	Meurens *et al.*, 2009
**MUC1**	S: TAAAGAAGACGGGCTTCTGGAS: CCGCTTTAAGCCGATCAAAC	60	134	XM_001926883
**MUC2**	S: ACCCGCACTACGTCACCTTCAS: GGCAGGACACCTGGTCATTG	62	150	Bruel *et al.*, 2009
**MUC4**	S: CTGCTCTTGGGCACTATATGAS: CCTGTGACTGCAGAATCAAC	60	133	DQ848681
**PBD-1**	S: ACCGCCTCCTCCTTGTATTCAS: CACAGGTGCCGATCTGTTTC	62	150	Meurens *et al.*, 2009
**PBD-2**	S: TTGCTGCTGCTGACTGTCTGAS: CTTGGCCTTGCCACTGTAAC	62	180	Meurens *et al.*, 2009
**PPAR gamma**	S: AAGACGGGGTCCTCATCTCCAS: CGCCAGGTCGCTGTCATCT	62	149	Bassaganya-Riera et al., 2006
**RPL-19**	S: AACTCCCGTCAGCAGATCCAS: AGTACCCTTCCGCTTACCG	60	147	Meurens *et al.*, 2009
**Secretory component**	S: ACTGGTGTCGCTGGGAAGAGAS: GACCGTGAAGGTGCCATTGC	64	131	CJ025705
**TGF beta**	S: GAAGCGCATCGAGGCCATTCAS: GGCTCCGGTTCGACACTTTC	64	162	Meurens *et al.*, 2009
**TNF alpha/**TNFSF2	S: CCAATGGCAGAGTGGGTATGAS: TGAAGAGGACCTGGGAGTAG	62	116	Meurens *et al.*, 2009
**TSLP**	S: AGGGCTTGTGCTAACCTACAS: ATCCGGCCTATCATCACAG	58	164	Meurens *et al.*, 2009

Reference genes are underlined.

**Table 2:** Effects of *Saccharomyces cerevisiae* or ETEC on transcript expressions in IPEC-1 cells (fold changes in comparison to controls).

Transcripts	Expression level	*S. cerevisiae*	ETEC
**APRIL**	moderate	0.60	1.13
**BAFF**	low	0.40 **	0.74
**CCL2**	moderate	1.32	6.83
**CCL17**	not detected	-	-
**CCL20**	moderate	3.61	5726.08 **
**CCL22**	not detected	-	-
**CCL25**	moderate	2.73**	1.11
**CXCL2**	moderate	1.65	857.44 **
**CXCL10**	moderate	1.18	7.05 **
**CXCL12**	moderate	1.50	2.98 **
**IFN-γ**	not detected	-	-
**IL-1α**	moderate	7.37	46.93 **
**IL-4**	not detected	-	-
**IL-5**	moderate	0.97	2.56 **
**IL-6**	high	0.39	7.80 **
**IL-8**	moderate	5.08	670.15 **
**IL-10**	low	1.07	2.00
**IL-12p35**	moderate	10.69 **	0.85
**IL-13**	low	0.93	0.93
**IL-17A**	not detected	-	-
**IL-23p19**	high	0.47	1.66
**IL-33**	not detected	-	-
**MUC1**	high	2.21 **	1.30
**MUC2**	low	1.46	0.74
**MUC4**	moderate	0.9	0.77
**PBD-1**	low	0.5	1.07
**PBD-2**	not detected	-	-
**PPAR-γ**	high	2.59 **	1.21
**Secretory component**	high	0.66	0.78
**TGF-β**	high	1.64	0.62
**TNF-α**	low	5.12	3075.63 **
**TSLP**	moderate	1.17	1.36

Level of mRNA expressions in untreated cells are expressed in the second column (high: Amplification around 17–24 *cq* (*cycle quantification*), moderate: Around 25–29 *cq*, low: Around 30–33 *cq*, not detected: More than 33 *cq*). Asterisks

** denote *p*<0.01

### *Saccharomyces cerevisiae* modulates the ETEC-induced transcript expression in differentiated IPEC-1 cells

**Figure 2:**
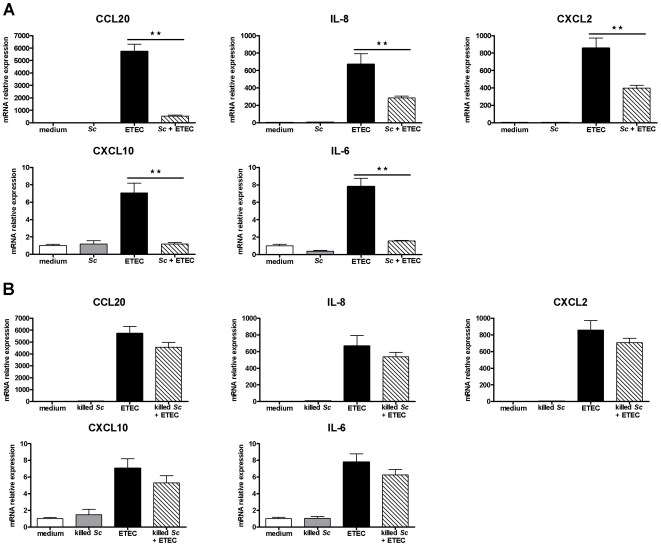
Viable *Saccharomyces cerevisiae* **down-regulates cytokine and chemokine mRNA relative expressions induced by ETEC.** Differentiated IPEC-1 cells were cultured overnight with (A) viable *Sc* or (B) killed *Sc* (3×10^6^ yeasts/well) and then ETEC (3×10^7^ CFU/well) was added to the co-culture for 3 h. Gene expression was analysed by RT-qPCR. Data are presented as means ± SEM (*n* = 6), asterisks denote: ** (*P<0.01*). (A) Data are representative of three independent experiments.

**Figure 3:**
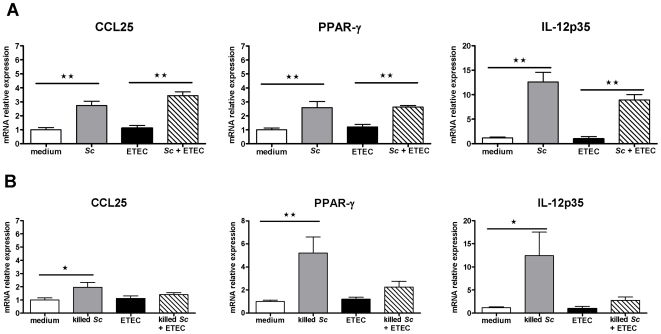
*Saccharomyces cerevisiae* **up-regulates CCL25, PPAR-γ and IL-12p35 mRNA relative expressions.** Differentiated IPEC-1 cells were cultured overnight with (A) viable *Sc* or (B) killed *Sc* (3×10^6^ yeasts/well) and then ETEC (3×10^7^ CFU/well) was added to the co-culture for 3 h. Gene expression was analysed by RT-qPCR. Data are presented as means ± SEM (*n* = 6), asterisks denote: * (*P<0.05*), ** (*P<0.01*). (A) Data are representative of three independent experiments.

Overnight pre-incubation of IPEC-1 cells with viable *Sc* before ETEC exposure for 3 h inhibited significantly CCL20 (÷ 10.7), CXCL10 (÷ 6), IL-6 (÷ 5), IL-8 (÷ 2.3), and CXCL2 (÷ 2.2) mRNA expressions *(p<0.01)* (Figure 2.A). In contrast, *Sc* did not decrease significantly the mRNA expressions of TNF-α, IL-1α, IL-5 and CXCL12 which were up-regulated by ETEC. In addition, *Sc* inhibitory effect on BAFF mRNA expression was not conserved in presence of ETEC (data not shown). However, when *Sc* was killed, no inhibitory effects were observed suggesting that yeast-secreted factors are essential to inhibit the ETEC-induced gene expressions (Figure 2.B). Regarding *Sc* stimulatory effects, overnight pre-incubation of IPEC-1 cells with viable yeasts still up-regulated IL-12p35, CCL25 and PPAR-γ mRNA expressions *(p<0.05)* in IPEC-1 cells exposed to ETEC for 3 h (respectively x 8.9, x 3.1 and x 2.2) (Figure 3.A). The MUC1 mRNA was still up-regulated in presence of ETEC as observed with *Sc* alone (x 2.0, *p<0.01*, data not shown). However, when the yeast was killed, no stimulatory effects were observed in presence of ETEC and only IPEC-1 cells cultured with killed *Sc* alone showed this pattern of up-regulated mRNA expression (Figure 3.B). Because *Sc* up-regulated CCL25 mRNA levels, we also analysed whether IPEC-1 cells expressed the homeobox transcription factors Cdx-1 and -2, which are involved in CCL25 transcription. Analysis displayed that in these conditions of co-culture, Cdx-1 and -2 mRNA were not expressed in differentiated IPEC-1 cells (data not shown).

### *Saccharomyces cerevisiae* decreases the ETEC-induced IL-6 and IL-8 secretions in differentiated IPEC-1 cells

**Figure 4:**
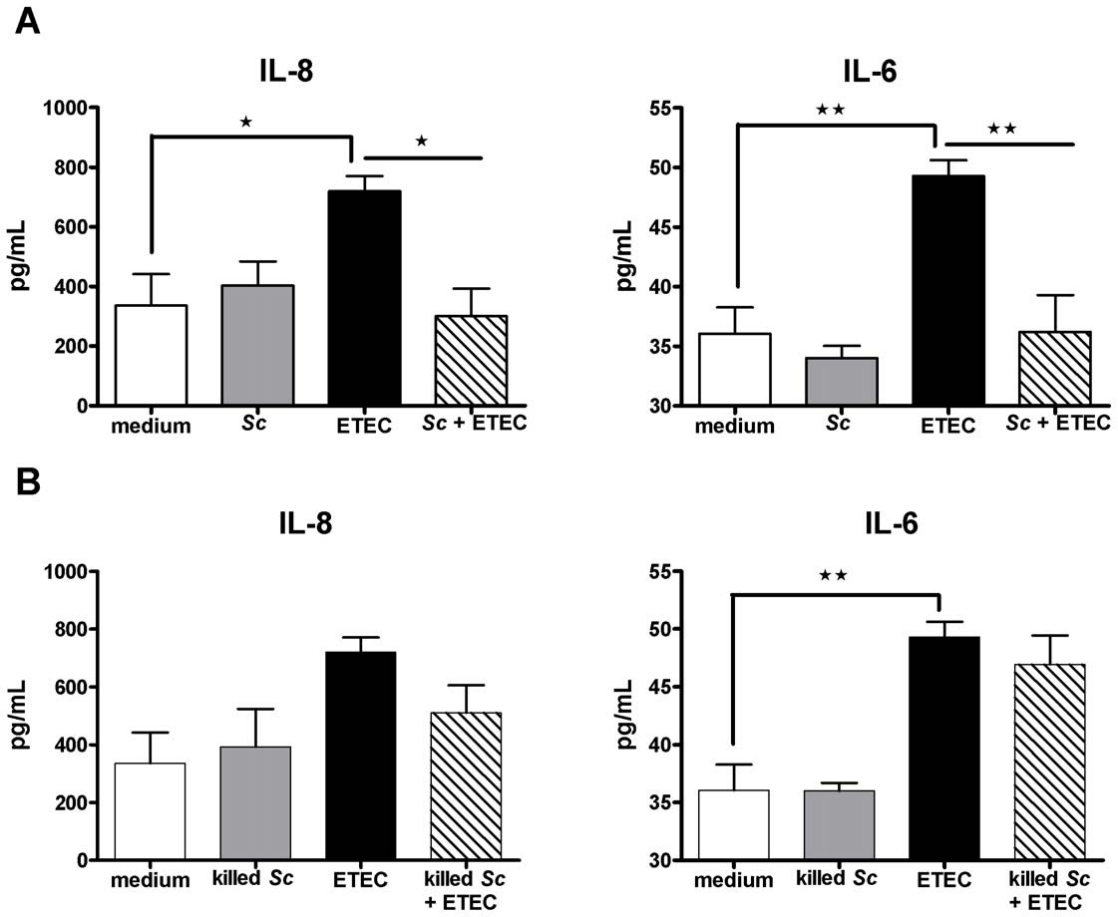
*Saccharomyces cerevisiae* decreases IL-6 and IL-8 secretions induced by ETEC. Differentiated IPEC-1 cells were cultured overnight with (A) viable or (B) killed *Sc* (3×10^6^ yeasts/well) and then ETEC (3×10^7^ CFU/well) was added to the co-culture for 30 min. Differentiated IPEC-1 cells were then washed and cultured for another 24 h before the assessment of apical IL-6 and IL-8 secretions by ELISA. Data are presented as means of cytokine concentration ± SEM (*n* = 3-4), asterisks denote: * (*P<0.05*), ** (*P<0.01*). Data are representative of two independent experiments.

At the protein level, overnight pre-incubation of IPEC-1 cells with viable or killed *Sc* did not modify apical IL-6 and IL-8 secretions in comparison to untreated cells (Figure 4.A, 4.B) whereas exposure with ETEC for 30 min up-regulated significantly the secretion of IL-6 (49.29±1.32 *versus* 36.06±2.21 pg/mL, *p<0.05*) and IL-8 (720±50.87 *versus* 335.8±105.8 pg/mL, *p<0.05*) (Figure 4.A, 4.B). In contrast, neither *Sc* nor ETEC induced the basolateral secretion of IL-6 and IL-8. The basolateral concentration of these cytokines was below the detection threshold of ELISA kits (data not shown). Overnight pre-incubation of IPEC-1 cells with viable *Sc* before ETEC exposure for 30 min inhibited significantly the apical secretions of IL-6 (36.21±3.10 *versus* 49.29±1.32 pg/mL, *p<0.01*) and IL-8 (300±92.21 *versus* 720±50.87 pg/mL, *p<0.05*) (Figure 4.A, 4.B). However, killed *Sc* did not inhibit significantly the ETEC-induced apical secretions of IL-6 and IL-8 (Figure 4.A, 4.B). These results are in accordance with those observed for the modulation of IL-6 and IL-8 transcripts indicating that *Sc* display anti-inflammatory properties at the molecular level.

### *Saccharomyces cerevisiae* decreases ERK1/2 and p38 MAPK phosphorylation in differentiated IPEC-1 cells

**Figure 5:**
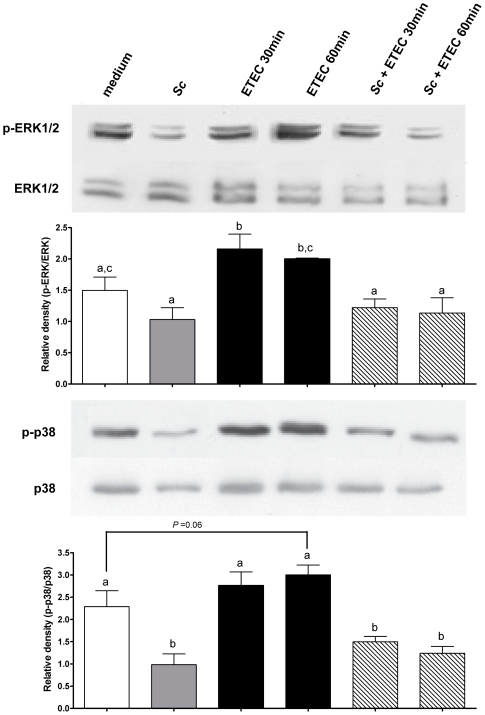
*Saccharomyces cerevisiae* decreases the MAP Kinase (ERK1/2, p38) phosphorylation in differentiated IPEC-1 cells. Differentiated IPEC-1 cells were exposed for 30 and 60 min with ETEC (3×10^7^ CFU/well) in the presence and absence of *Sc* (3×10^6^ yeasts/well). Western blots for phospho-ERK1/2 (p-ERK1/2) and phospho-p38 (p-p38) were performed. Total ERK1/2 and total p38 are shown as loading controls and did not change with each condition over time. Data are presented as means ± SEM, (*n* = 3) and the different letters represent significant differences between the treatments (*P<0.05*). Results are representative of three independent experiments.

We next examined the effect of *Sc* on the activation of different protein kinases which are involved in pro-inflammatory gene expressions. Thus, we analysed *Sc* modulatory effects on MAPK (ERK1/2, p38, JNK), Akt and AMP-activated protein kinase (AMPK) phosphorylation using phospho-specific antibodies. As shown in Figure 5, *Sc* decreases the phosphorylation of ERK1/2 and p38. In control cells, the phosphorylated active-forms of ERK1/2 and p38 are detectable. Cells pre-incubated with *Sc* alone showed a significant decrease in p38 phosphorylation *(p<0.05)* but not in ERK1/2 (Figure 5). In contrast, ETEC exposure for 30 min enhanced the phosphorylation level of ERK1/2 *(p<0.05)* and ETEC exposure for 60 min enhanced the phosphorylation of p38 *(p = 0.06)* (Figure 5). When IPEC-1 cells were pre-incubated with *Sc* and then infected with ETEC, the active form of ERK1/2 was reduced at the level of control cells *(p<0.05)* whereas the active form of p38 was lower than control cells *(p<0.05)* (Figure 5). No effect was observed on Akt and AMPK activation, and JNK detection level was too low to display any regulatory effect of *Sc* (data not shown). These results indicated that *Sc* inhibitory effects on cytokine and chemokine mRNA expressions are associated with a decrease of ERK1/2 and p38 phosphorylation in IPEC-1 cells.

### *Saccharomyces cerevisiae* agglutinates ETEC

**Figure 6:**
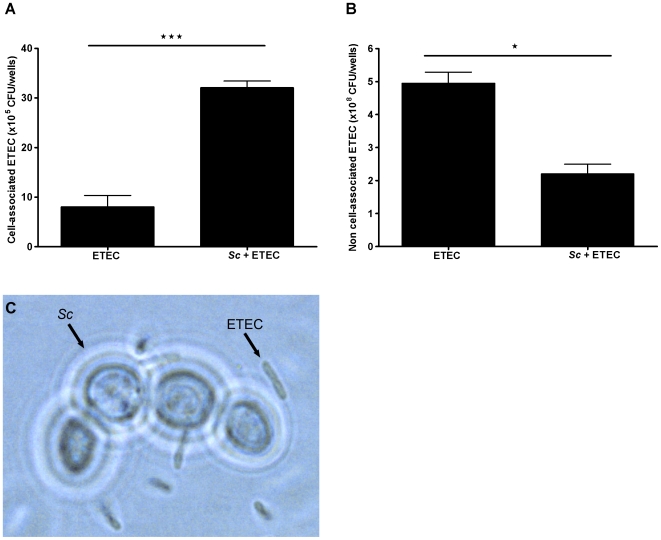
*Saccharomyces cerevisiae* agglutinates ETEC. (A) IPEC-1 cells were exposed to ETEC (3×10^7^ CFU/well) for 30 min in the presence or absence of *Sc* (3×10^6^ yeasts/well). Cells were then lysed and cell lysates were diluted and plated on agar in order to quantify the cell-associated bacteria, (*n* = 4). (B) IPEC-1 cells were exposed to ETEC (3×10^7^ CFU/well) for 3 h in the presence or absence of *Sc* (3×10^6^ yeasts/well) and 100 µl of culture supernatant were then harvested from the apical compartment, diluted and plated on agar in order to quantify the non cell-associated bacteria, (*n* = 4). (C) IPEC-1 cells were overnight pre-incubated with *Sc* (3×10^6^ yeasts/well) and then exposed to ETEC (3×10^7^ CFU/well) for 3 h. Apical IPEC-1 cell culture supernatant was harvested and physical interaction between *Sc* and ETEC was observed by phase contrast microscopy (x 1000). Data are presented as means ± SEM, asterisks denote: * (*P<0.05*), *** (*P<0.001*). (A, B) Data are representatives of two independent experiments.

We investigated whether *Sc* modulates ETEC growth and adhesion on IPEC-1 cells. Overnight pre-incubation of IPEC-1 cells with *Sc* before ETEC exposure increased the number of cell-associated ETEC (8.02±2.31×10^5^ CFU/wells in ETEC group *versus* 32.08±1.33×10^5^ CFU/wells in *Sc*+ETEC group) and decreased the number of non cell-associated ETEC (4.95±0.34×10^8^ CFU/wells in ETEC group *versus* 2.2±0.29×10^8^ CFU/wells in *Sc*+ETEC group) (Fig. 6.A, 6.B). Consequently, we assessed whether adherent yeasts on IPEC-1 cells could interact with ETEC thus forming agglutinates on the surface monolayer. Scanning electron microscopy of IPEC-1 cells exposed with ETEC did not demonstrate ETEC agglutination by *Sc* on the surface monolayer. In contrast, a physical interaction between yeasts and ETEC isolated from IPEC-1 cells culture supernatant was observed by phase-contrast microscopy (Fig. 6.C).

### *Saccharomyces cerevisiae* did not prevent the disruption of membrane barrier integrity induced by ETEC

**Figure 7:**
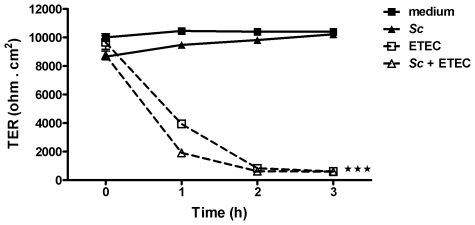
*Saccharomyces cerevisiae* failed to preserve the barrier function. Measurement of transepithelial electrical resistance (TER) in differentiated IPEC-1 cells untreated, pre-incubated with *Sc* (3×10^6^ yeasts/well), exposed to ETEC (3×10^7^ CFU/well) or pre-incubated with *Sc* and then exposed with ETEC. Data are presented as means ± SEM, (*n* = 4). Data are representative of two independent experiments, asterisks denote: *** (*P<0.001*).

To assess the protective effect of *Sc* on the monolayer barrier function, transepithelial electrical resistance (TER) was measured in IPEC-1 cells pre-incubated or not with *Sc* and then exposed to ETEC for 3 h. Overnight pre-incubation of IPEC-1 cells with *Sc* alone did not alter the monolayer resistance (TER>8000 ohm.cm^2^) (Figure 7). In contrast, when IPEC-1 cells were exposed to ETEC, a significant TER decrease was already observed at 1 h of exposure (3934±50 ohm.cm^2^, *p<0.001*) (Figure 7). Overnight pre-incubation of IPEC-1 cells with *Sc* before ETEC exposure did not preserve the barrier function in comparison to both untreated and *Sc*-treated cells, thus resulting in a significant TER decrease after 1 h of ETEC exposure (1915±246 ohm.cm^2^, *p<0.001*) (Figure 7). In presence of *Sc*, despite a higher TER drop at 1 h of ETEC exposure, no differences were observed at 2 h and 3 h in comparison to monolayers exposed with ETEC alone (Figure 7).

## Discussion

In pigs, enterotoxigenic *Escherichia coli* (ETEC) is the most common etiologic agent of enteric diseases in the weaning period [1]. ETEC infection induces pro-inflammatory responses in porcine intestinal epithelial cells [37] and causes diarrhea resulting in reduced growth rate, mortality and economic loss [1], [5]. Since 2006, administration of antibiotics as growth promoters in the piglet diet was prohibited in the European Union (IP/05/1687) and consequently, alternative strategies such as probiotics were developed to prevent intestinal diseases and to maintain health status [38], [39].

In this study, we investigated *Saccharomyces cerevisiae* (*Sc*, strain CNCM I-3856) modulatory effects on the expression of genes involved in inflammation, recruitment and activation of immune cells. IEC represent a relevant model to study yeast probiotic effects in the intestinal tract because IEC are involved in several mechanisms allowing protection such as barrier function, mucus layer protection, antibacterial peptide secretion and cytokine and chemokine secretion [19]. Consequently, IEC are involved in innate immunity as well as in the induction of adaptive immunity at the mucosal surface. In this study, we used as model of IEC the porcine IPEC-1 cell line exposed to ETEC. Previous reports suggest the presence of F4 receptor onto the apical surface of IPEC-1 cells since ETEC F4^+^ can adhere to these cells [34], [35]. In addition, IPEC-1 exposure to ETEC F4^+^ stimulate pro-inflammatory responses in IEC [35], [37]. Moreover, bacterial constituents like flagellin and lipopolysaccharide have been shown to activate MAPK signaling pathways in IEC [40], [41]. In the current work, we showed that only viable *Sc* decreased the ETEC-induced mRNA expressions of pro-inflammatory IL-6, IL-8, CCL20, CXCL2 and CXCL10. In addition, viable *Sc* decreased the apical secretions of IL-6 and IL-8 whereas no secretions were observed at the basolateral level. This result indicates a polarization of IL-6 and IL-8 secretions as described previously in both polarized epithelial and endothelial cells [42], [43], [44]. Taken together, these results indicate that *Sc* viability is essential and confirm the presence and the action of a soluble secreted factor as demonstrated in previous studies [45], [46]. In addition, a recent study reported that *Sc* (strain CNCM I-3856) exerts antagonist effect on *E. coli* O157:H7 probably through ethanol production [33], thus highlighting another potential anti-inflammatory effect used by the yeast. In our study, inhibition of pro-inflammatory gene expressions was correlated with a decrease of the MAPK ERK1/2 and p38 phosphorylation. These results are reminiscent of other studies showing that *Sb* inhibits IL-8 expression and MAPK phosphorylation induced by enteric pathogens such as enterohemorrhagic *E. coli* [21], *Clostridium difficile* [20], *S. enterica* Serovar Typhimurium [18] and *Shigella flexneri* [16]. Inhibition of pro-inflammatory IL-6, CCL20 and CXCL10 transcript expressions could also be associated with the inhibition of ERK1/2 and p38 MAPK signaling pathways since the activation of these protein kinases has been shown to correlate with IL-6, CCL20 and CXCL10 expressions in different cell types [47], [48], [49], [50]. In contrast, detection level of JNK in untreated and ETEC-exposed IPEC-1 cells was too low to display any regulatory effect of *Sc*. We also analysed whether or not the inhibition of the pro-inflammatory responses might be due to a modulation of Akt and AMPK activity. Previous studies described that Akt and AMPK are activated during intestinal inflammation [51], [52]. Moreover, *Sb* has been shown to decrease Akt activation in human HT29 colonocytes [53]. Neither *Sc* nor ETEC modulated Akt and AMPK signaling pathways in our model. This result could be explained by their activation at a different time point. In addition, *Sc* and ETEC could stimulate pathogen recognition receptors which did not activate these protein kinases in IPEC-1 cells. Taken together, these results show that *Sc* (strain CNCM I-3856) interfer with MAPK (ERK1/2, p38) signaling pathways and decrease pro-inflammatory responses in porcine intestinal epithelial IPEC-1 cells exposed with ETEC. These anti-inflammatory properties are in accordance with those described with the probiotic yeast *Sb* [16], [18], [20] and indicate that both *Sc* and *Sb* can inhibit cell signaling pathways despite their genetic, metabolic and physiologic differences [27], [28]. Several reports displayed also that *Sb* decrease NF-κB activation in IEC infected with enteric pathogens such as enterohemorrhagic *E. coli*, *S. flexneri* and *S. enterica* [16], [18], [22]. However, *Sc* effect on NF-κB activation was not assessed in this study and thus requires further investigations.

In addition, we showed that *Sc* up-regulated significantly the mRNA levels of the pro-Th1 cytokine IL-12p35, the chemokine CCL25 and the nuclear receptor PPAR-γ. CCL25 is known to be involved in T and B cells recruitment into the small intestine [54], [55]. Thus, *Sc* might stimulate the mucosal immune response and IgA plasma cell recruitment through enhancement of CCL25 expression. Previous *in vivo* studies displayed that oral administration of *Sb* to mice increases the concentration of intestinal IgA [17], [56]. The up-regulation of CCL25 expression could potentially explain, at the molecular level, one mechanism used by probiotic yeasts to stimulate the mucosal immune response. Because IPEC-1 cells express CCL25, we also assessed the expression of Cdx-1 and -2 transcription factors which has been shown to regulate CCL25 transcription [57]. Our study did not show any Cdx-1 and -2 transcript expressions by IPEC-1 cells. This result is in agreement with previous studies reporting that Cdx-1 and -2 were not expressed by immortalized epithelial cell lines [57], [58] suggesting either a different mechanism for CCL25 regulation or an activation of Cdx-1 and -2 expression at a different time point. *Sc* increased the transcript expression of IL-12p35 whereas ETEC did not. This result is in accordance with previous studies showing that *Sc* stimulates the secretion of IL-12p70 in activated human neutrophil-like 60 cells and monocyte-derived dendritic cells [59], [60]. In contrast, *E. coli* pathovars such as ETEC failed to stimulate IL-12p40 expression in bovine primary colonocytes [61]. These results may suggest that *Sc* and ETEC could influence differently the immune response in part through modulation of IL-12 expression level. PPAR-γ is a nuclear receptor expressed by several cell types including IEC, dendritic cells, T and B cells and acts as a regulator of the inflammation [62], [63], [64]. In our study we shown an up-regulation of PPAR-γ transcripts and thus *Sc* could also mediate anti-inflammatory effects through activation of PPAR-γ. This result could be correlated with previous studies showing that *Sb* up-regulated PPAR-γ expression in human colonocytes [65] and that PPAR-γ decreased pro-inflammatory cytokine levels in IEC [66]. Moreover, previous studies showed that activation of ERK1/2 regulated negatively the expression of PPAR-γ [67], [68] suggesting that both *Sc* stimulatory effects on PPAR-γ expression and inhibitory effects on ERK1/2 activation could be linked [69].

In this study, we also showed that the number of cell-associated ETEC is increased in presence of *Sc* while the number of non cell-associated ETEC is decreased. Consequently, we hypothesized that adherent yeasts on IPEC-1 cells could agglutinate ETEC thus forming agglutinates on the surface monolayer as shown previously with *S. enterica* serovar Typhimurium on the surface of T84 cells [18]. An agglutination of ETEC by *Sc* was observed by phase-contrast microscopy in IPEC-1 cell culture supernatant. This result suggests that the higher number of cell-associated bacteria observed in presence of *Sc* might be due to yeast-bacteria agglutinates on the surface monolayer despite the lack of confirmation by scanning electron microscopy. Furthermore, the agglutination of ETEC by yeasts may explain partially the inhibition of pro-inflammatory gene expressions in IPEC-1 cells which could be less stimulated by bacterial components such as LPS or flagellin. We also determined that ETEC decreased significantly the TER of IPEC-1 cells exposed to ETEC. This result is in agreement with others reports showing that ETEC decreased the TER in porcine IPEC-1 and IPEC-J2 cells [70], [71] and altered tight-junction structure in IPEC-1 cells [35]. TER measurement displayed that *Sc* failed to preserve the barrier function of infected IPEC-1 cells despite its anti-inflammatory activity. This result differed from a previous report showing that yeast extracts prevent both TER and membrane permeability decreases induced by ETEC [71]. However, previous studies suggested that TER decrease may reflect alterations in ion channel function [72], [73]. Consequently, this result suggests that *Sc* does not inhibit ETEC enterotoxin activity leading to the disruption of the transepithelial resistance.

In conclusion, results demonstrate that *Sc* (strain CNCM I-3856) inhibits pro-inflammatory gene expressions. This inhibition is associated to the modulation of both ERK1/2 and p38 signaling pathways, the increase of PPAR-γ transcript expression and the ETEC agglutination by yeasts. *Sc* stimulates also the transcript expression of CCL25 and IL-12p35. These results indicate that *Sc* exerts immuno-modulatory effects at the molecular level in IPEC-1 cells encouraging the assessment of *Sc in vivo* modulatory effects in the immune and inflammatory responses.

## Materials and Methods

### Epithelial cell line culture

The non-transformed porcine intestinal epithelial cell line IPEC-1 was derived from the small intestine of a newborn unsuckled piglet [74]. Cells were cultured in DMEM/F-12 medium (Invitrogen, Cergy Pontoise, France) supplemented with 5% foetal calf serum (Sigma–Aldrich, Saint-Quentin, France), 2 mM L-glutamine (Invitrogen), 1% insulin-transferrin-selenium (Sigma-Aldrich, Saint-Quentin, France), 10 ng/ml epidermal growth factor (Invitrogen), 50 U/ml penicillin and 50 µg/ml streptomycin (Invitrogen). For co-culture experiments, IPEC-1 cells were seeded onto 4.2 cm^2^ cell culture inserts (pore size 0.4 µm, Becton Dickinson Labware, Le Pont De Claix, France) at 3.5×10^5^ cells per inserts. Inserts were not collagen-coated. IPEC-1 cells were grown for 2 days until 100% of confluence and then differentiated for 10 days. For differentiation culture, the medium described above was modified with the omission of foetal calf serum and the addition of 10^-7^ M dexamethasone (Sigma–Aldrich). Differentiation culture medium was changed every 2 days. Once differentiated, IPEC-1 cells had an average cell density of 1×10^6^ cells/well and the transepithelial electrical resistance measured with a Millicell-ERS volt-ohm meter (Millipore, Molsheim, France) was typically around 8000 ohm.cm^2^. Differentiated IPEC-1 cells exhibit apical and basolateral surfaces, form apical microvilli and express tight junction proteins [35], [74].

### Microorganisms' growth

The ETEC strain GIS26 (O149:K91, F4ac^+^, LT^+^ STa^+^ STb^+^ : H19) was grown in Luria-Bertani (LB) medium containing 1% tryptone, 0.5% yeast extract, 1% NaCl, pH 7. After overnight incubation at 37°C with vigorous shaking, bacteria were diluted at 1∶400 in fresh LB and grown until midlog phase (∼4 h) for all experiments.

*Saccharomyces cerevisiae* (strain CNCM I-3856, Lesaffre yeast collection, CNCM: French National deposit Collection of Microorganism Cultures, Institut Pasteur, Paris, France) were provided by Lesaffre (Société industrielle Lesaffre, Marcq-en-Baroeul, France) as a dry form at a concentration of 1×10^10^ yeasts/g. *Sc* was rehydrated in free-DMEM/F-12 medium (Invitrogen, Cergy Pontoise, France) for 45 min at 30°C. For the different experiments, concentration of viable *Sc* was established by methylene blue exclusion (0.3 g/L methylene blue, 20 g/L sodium citrate). To evaluate the effects of killed *Sc*, yeasts were rehydrated in DMEM/F-12 medium and then frozen in liquid nitrogen. After ten cycles of freezing/thawing, mortality resulted in 100% of killed yeasts.

### Antibodies

Immunoblots were performed using the phosphorylated antibodies: (p)-ERK1/2 (4377S, Ozyme, Saint-Quentin-en-Yvelines, France), p-p38 (9211S, Ozyme), p-JNK 1/2/3 (9251, Ozyme), p-AMPK (2535L, Ozyme), p-Akt (sc-7985-R, Santa Cruz Biotechnology, Heidelberg, Germany), and the total antibodies: ERK2 (sc-154, Santa Cruz Biotechnology, cross-reacts with ERK1), p38 (sc-535, Santa Cruz Biotechnology), JNK (9252, Ozyme), AMPK (2532L, Ozyme), Akt (9272, Ozyme). According to the manufacturers, all antibodies react with human whereas only antibodies specific for p-ERK1/2, p-p38 and Akt cross-react with pig.

### IPEC-1 exposure to ETEC

Prior to exposure, IPEC-1 cells (∼1×10^6^ cells/well) were washed three times in medium without serum and antibiotics. The ETEC strain GIS26 was grown for 4 h in LB medium, pelleted by centrifugation at 2000 g for 20 min, resuspended in sterile phosphate-buffered saline and added to IPEC-1 cells at 3×10^7^ CFU/well (30 bacteria/cell) for 3 h. When experiments were performed in presence of *Sc*, IPEC-1 cells were pre-incubated overnight with 3×10^6^ yeasts/well (3 yeasts/cell) and then exposed to ETEC for 3 h. Overnight pre-incubation of IPEC-1 cells with *Sc* was chosen in order to assess the yeast preventive effects as described previously [22], [23]. Both ETEC and *Sc* were added to the apical compartment.

### Analysis of relative mRNA expression using quantitative real-time PCR

IPEC-1 cells (∼1×10^6^ cells/well) were incubated overnight with viable or killed *Sc* (3×10^6^ yeasts/well) and then exposed to ETEC (3×10^7^ CFU/well) for 3 h. IPEC-1 cells were lyzed with Trizol reagent (Invitrogen, Cergy-Pontoise, France) and total RNA was isolated using RNeasy Mini Kit (Qiagen, Courtaboeuf, France). Quantitative real-time PCR (qPCR) was performed using cDNA synthesized as previously described [58]. Diluted cDNA (10X) was combined with primer/probe sets and MESA GREEN qPCR MasterMix (Eurogentec, Liège, France) according to the manufacturer's recommendations. The qPCR conditions were 95°C for 30 s, followed by 37 cycles with denaturation at 95°C for 15 s and annealing/elongation for 45 s. To minimize sample variation, we used high quality RNA. Samples were normalized internally using simultaneously the average cycle quantification (Cq) of Hypoxanthine PhosphoRibosyl-Transferase 1 (HPRT-1), Ribosomal Protein L 19 (RPL-19) and Hydroxymethylbilane synthase 2 (HMBS2) [75] as references in each sample to avoid any artefact of variation in the target gene. These reference genes were selected for their stable expression in IPEC-1 cell line as described previously [76]. The primer sequences and the annealing temperatures are described in Table 1. Real time assays were run on a Bio-Rad Chromo4 (Bio-Rad, Hercules, CA, USA). Expression data are expressed as relative values after Genex macro analysis (Bio-Rad, Hercules, CA, USA) [77]. The design of qPCR experiments and the analysis of results were performed following the MIQE guidelines [78].

### Measurement of apical IL-6 and IL-8 production

IPEC-1 cells (∼1×10^6^ cells/well) were incubated overnight with viable or killed *Sc* (3×10^6^ yeasts/well) and then exposed to ETEC (3×10^7^ CFU/well) for 30 min. The time of ETEC exposure was chosen to avoid a massive alteration of the monolayer after the subsequent washes. After 3 washes, differentiation culture medium containing 50 µg/mL of gentamycin (Invitrogen) was added on IPEC-1 cells for 24 h. Apical and basolateral supernatants were then removed and cytokine production was measured by ELISA using commercial kits (R&D for IL-6 assay and Invitrogen for IL-8 assay).

### Western Blotting

IPEC-1 cells (∼1×10^6^ cells/well) were incubated overnight with *Sc* (3×10^6^ yeasts/well) and then exposed to ETEC (3×10^7^ CFU/well) for 30 and 60 min. Cells were then lysed in TNET lysis buffer (20 mM Tris pH 7.8, 50 mM NaCl, 5 mM EGTA, 1% (v/v) Triton X-100, 1 mM phenylmethylsulfonyl fluoride, 4 mM Na_3_VO_4_, 5 µg/ml leupeptin, 5 µg/ml pepstatin, and 5 µg/ml aprotinin). Equal amounts of proteins were separated on SDS-PAGE and transferred onto a nitrocellulose membrane. Membranes were incubated for 1 h at room temperature with Tris-buffered saline (TBS, 2 mM Tris–HCl, pH 8.0, 15 mM NaCl, pH 7.6), containing 5% non fat dry milk powder (NFDMP) and 0.1% Tween-20 to saturate non specific sites. Then, membranes were incubated overnight at 4°C with appropriate primary antibodies (final dilution 1∶1000) in TBS containing 0.1% Tween-20 and 5% NFDMP. After washing in TBS–0.1% Tween-20, the membranes were incubated for 2 h at room temperature with a HRP-conjugated goat anti-rabbit secondary antibody (final dilution 1∶10000; Diagnostic Pasteur, Marnes-la-Coquette, France) in TBS–0.1% Tween-20. After washing in TBS–0.1% Tween-20, the signal was detected by ECL (ECL, Amersham Pharmacia Biotech, Orsay, France). The films were analysed and signals quantified with the software Scion Image (4.0.3.2 version; Scion Corporation, Frederick, MD, USA).

### *Saccharomyces cerevisiae* effects on ETEC adhesion to IPEC-1 cells

The number of cell-associated ETEC was determined by agar plating. IPEC-1 cells (∼1×10^6^ cells/well) were infected with ETEC (3×10^7^ CFU/well) for 30 min in the presence or absence of *Sc* (3×10^6^ yeasts/well). The time of ETEC exposure was decreased from 3 h to 30 min to avoid a massive alteration of the monolayer after the subsequent washes. After 6 washes, cells were lysed with ultra pure water supplemented with 1%-Triton-X-100. Cell lysates containing the cell-associated ETEC were diluted and plated on 5% sheep blood agar. After 20 h of growth at 37°C, the number of ETEC colony was determined.

The number of non cell-associated ETEC was determined by agar plating. IPEC-1 cells (∼1×10^6^ cells/well) were infected with ETEC (3×10^7^ CFU/well) for 3 h in the presence or absence of *Sc* (3×10^6^ yeasts/well). Then, 100 µl of culture supernatant were harvested from the apical compartment, diluted and plated on 5% sheep blood agar. After 20 h of growth at 37°C, the number of non cell-associated ETEC was quantified.

### Scanning electron microscopy and phase contrast microscopy

For scanning electron microscopy analysis, IPEC-1 cells (∼1×10^6^ cells/well) were incubated overnight with *Sc* (3×10^6^ yeasts/well) and then exposed to ETEC (3×10^7^ CFU/well) for 45 min. This time was chosen to avoid a massive alteration of the monolayer induced by the subsequent washes. Then, IPEC-1 cells were washed 3 times in PBS and then fixed in a mixture of 4% paraformaldehyde and 1% glutaraldehyde in phosphate buffer (0.3 M; pH 7.4) for 1 h. IPEC-1 cells were washed with phosphate buffer pH 7.4 with 0.4% NaCl (w/v) and dehydrated through a graded series of 50%, 70%, 90% and 100% alcohol solution and HMDS (hexa-methyl-disilazan). Then, they were let dried and coated with a thin layer of platinium on a PECS Gatan coater. Samples were observed on Zeiss Ultra+FEGSEM scanning electron micrograph (Carl Zeiss S.A.S, Le Pecq, France).

For phase contrast microscopy, IPEC-1 cells (∼1×10^6^ cells/well) were incubated overnight with *Sc* (3×10^6^ yeasts/well) and then exposed to ETEC (3×10^7^ CFU/well) for 3 h. The apical cell culture supernatant was then removed and the interaction between *Sc* and ETEC was observed by phase contrast microscopy (Olympus BX51, Olympus S.A.S, Rungis, France).

### Transepithelial electrical resistance measurements

Transepithelial electrical resistance (TER) was measured to assess the integrity of epithelial monolayers using a Millicell-ERS volt-ohm meter (Millipore, Molsheim, France). IPEC-1 cells cultured onto 4.2 cm^2^ cell culture inserts were pre-incubated overnight with *Sc* (3×10^6^ yeasts/well) and then exposed to ETEC (3×10^7^ CFU/well) for 3 h. TER was measured each hour after ETEC addition.

### Statistical analysis

The comparison of the differences in mRNA relative expression and cytokine production were evaluated by one-way ANOVA and differences tested by non-parametric Dunnett's test. (using GraphPad Prism software version 4.00, GraphPad Software Inc., San Diego, CA, USA). Differences were considered significant when *P<0.05*.
